# The foundational role of autonomy for brain-computer interfaces

**DOI:** 10.1186/s13010-026-00231-4

**Published:** 2026-09-21

**Authors:** Florian Richter

**Affiliations:** School of Transformation and Sustainability, Catholic University of Eichstätt-Ingolstadt, Eichstätt, Germany

**Keywords:** Brain-computer interfaces, Autonomous systems, Human agency

## Abstract

Invasive brain-computer interfaces (BCIs) pose a challenge for describing human-system interactions because they involve a depth of engagement that is difficult to disentangle. This is particularly problematic when clear authorship of decisions is needed. This presupposes that such devices might also decide autonomously in certain situations. Although the use and implementation of such devices are highly disputed in practical ethics, they seem to offer at least therapeutic benefits for persons with, e.g., Parkinson’s disease. That is why it is indispensable to develop a conceptual framework that allows a description of the system’s interactions with the human agent. The effects of brain-computer interfaces on patients are assessed empirically in studies and hypothesized on a more speculative level via thought experiments. A model to operationalize dimensions of agency, such as responsibility and privacy, has been proposed. Nevertheless, this model is inchoate if it is not related to autonomy, because it is widely assumed that autonomy is not only a characteristic of the human agent but also of the technological system (at least to a certain degree). Consequently, a more complete model is needed that classifies autonomy at different levels (operational, strategic, and moral). Technological systems can only operate on the first two levels because their behavior is based on dispositions within the scope of alethic modality, whilst human agency is based on deontic structures that are expressed via normative attitudes and statuses that cannot be reduced to alethic modality. This crucial difference can be used to develop methods for evaluating the ethical side of BCIs.

## Introduction

Human-system interaction is mediated by interfaces, which are typically keyboards, screens, mice, and speech and text inputs and outputs. Other types of interactions can also be between humans and virtual agents or robots (for an overview, see Koch, et al. [30]). Although certain artifacts that monitor or track users are often embedded in the environment and thus might not be visible to the user, it is generally possible to identify the device and determine which data it tracks. This is different in the case of brain-computer interfaces (BCIs) that have – depending on the device and method – a depth of engagement in the human brain that cannot be disentangled easily. The use of BCIs follows different objectives that range from therapeutic and medical applications [46] to possible new forms of communication with computers and with others via such technological devices. Li and Zhang, [32] This is driven by many advancements in neurotechnology, such as correlating a stimulus (e.g., a picture) with specific brain activity using functional magnetic resonance imaging (fMRI) Kay et al. [28].

Despite such advancements, the blurring of a clear distinction between the human agent and the system poses challenges. Such obliteration can be entailed in three ways:

1. The technology is integrated into the lifeworld and is ready-to-hand so that it no longer appears as an object or artifact.
2. Sensors and other technological devices are embedded into the environment in a way that they are not visible or identifiable to humans who move and act within this smart environment.
3. The technological device is enmeshed with the human, such as chips or electrodes in the brain.

All three conditions need conceptual clarifications to improve our understanding of technology and how we use it. Still, the last one is the more drastic because, in the first two, it is always possible (at least to a certain degree) to abstain from using the device, take it away, or distance oneself from it. In the case of invasive brain-computer interfaces, it might be possible to turn the device off; nevertheless, the surgical implementation is not easily reversible. Even for noninvasive therapies, it is of theoretical concern that there might be “permanent changes in the brain” and unintended side effects that are also not reversible. (Cabrera et al. [11], p. 37/38) Consequently, BCIs represent a paradigmatic and borderline case of human-system interaction that needs careful scrutiny.

### Technical and medical background

Brain-computer interfaces can be noninvasive, such as electroconvulsive therapy (ECT) (Belge, et al. [4]), transcranial magnetic stimulation (TMS) (Janicak & Dokucu [25]), or theta burst stimulation (TBS) (Suppa, et al. [48]), to treat major depressive disorders. However, an ECT might not require a computerized system, so there is no communicative interaction between the device and the brain. Here, the ethical concerns will be instead on invasive BCIs, which include deep brain stimulation (DBS) (Thomsen, et al. [50], Limousin & Foltynie, [33] or adaptive DBS (Swann, et al. [49]) to treat Parkinson’s disease. However, whether some kind of brain stimulation is taken as BCIs or as a subgroup of them depends on the meaning of “interface.” If interfacing means enabling communication through artifacts such as screens or keyboards, then definitely not; brain-computer interfaces are usually understood as connecting the human brain to some kind of technical artifact or system (a stimulator via electrodes or a chip).^1^ Specifically, the confusion about whether an interface can entail an interaction stems from the ambiguity of the term “interface.” It arises when it is not clarified which level of autonomy the artifact or system has. This will be analyzed in section three.

Schönau et al. summarize some of the benefits of the applications in the medical field:

- For a “person with Parkinson’s disease,” the “benefit from a deep brain stimulator (DBS)” may be that it “alleviates tremor and rigidity, and thus restores the ability to fluently perform movements.”
- For a “person living with spinal cord injury,” the “benefit from a brain computer interface (BCI)” might be “to control a robotic arm, or even to regain a lost sensation of touch.”
- For a “person with amyotrophic lateral sclerosis (ALS),” the benefit from the use of a BCI may be “to communicate with loved ones through the translation of thought to computer generated speech.”
- A person with depression “may use a DBS to improve mood, in the hope of regaining a brighter, more authentic self.” (Schönau, et al. [43], p. 172).

The therapeutic and medical aspects will be discussed further in section three to understand what is possible technology-wise and to analyze the implications regarding the levels of autonomy.

### Ethical background

Although it is argued by some “that BCI users perceive themselves as ‘active operators’ who recognize and appreciate a neural device’s capacity to maintain or restore human capabilities,” Schönau et al. state that “the neuroethics literature is replete with examples of end users expressing concerns, such as a sense of alienation or a perceived lack of control.” (Schönau, et al. [43], p. 172) Thus, the results, analyzed from an ethical perspective, are highly questionable despite the promises that the advancements in the therapeutic or medical field seem to offer, i.e., to cure or at least alleviate symptoms of certain diseases. The depth of engagement is much higher in brain-computer interfaces than in other forms of interactions with technological systems. As a means of addressing this problem, Yuste et al., proposed “four areas of concern” for ethical discussions (“privacy and consent,” “agency and identity,” “augmentation,” and “bias”). (Yuste, et al. [55]) Goering et al., identify similar “ethical challenges” (“identity and agency, privacy, bias, and enhancement”). (Goering, et al. [16], p. 365) Nonetheless, neither Yuste et al., nor Goering et al., discuss autonomy as a guiding concept. (Yuste, et al. [55]), (Goering, et al. [16]) The “OECD Recommendation on Responsible Innovation in Neurotechnology” emphasizes autonomy several times, but in a rather abstract and general way. OECD, [38] Rommelfanger et al., relate autonomy to “control,” “free will,” and authorship of actions. However, they state that there is no consensus in the research community on how to conceive autonomy. (Rommelfanger, et al. [42], p. 30) In contrast, Cabrera et al., and Greely et al., discuss autonomy with regard to “informed consent” and “identity/personhood.” (Cabrera et al. [11], pp. 38–41), (Greely, et al. [18], p. 10587) However, such aspects cover only parts of the concept of autonomy. This will be discussed further below (2.3). Another approach is from Schönau et al., who address four ethical dimensions (“responsibility,” “privacy,” “authenticity,” and “trust”) and their relation to agency. (Schönau, et al. [43]) Still, such concepts need to be related to the concept of autonomy, which serves as the conditioning factor for understanding the ethical issues of BCIs. If a person is no longer able to grasp herself as the originator of the action, she will also not feel responsible for her action, i.e., she will take the action not as being part of her authentic self or identity. That is why an amplified model based on different types of autonomy is proposed here. It will also allow for a better description of the normative elements of human agency.

This paper makes four contributions.^2^ First, it analyzes how ethical questions regarding BCIs must be linked to agency; otherwise, they will not be empirically testable or operationalizable. Second, it is shown that autonomy is foundational to conceptualizing agency; otherwise, agency would be reduced to mere behavior. Third, different conceptions or models of autonomy are discussed. Most conceptions either differentiate autonomy across various fields of application (e.g., political and personal autonomy) or explain it in terms of various aspects (e.g., self-control, self-determination, informed consent, identity, personhood, etc.). However, levels of autonomy have not yet been introduced into or applied to the field of neuroethics. The conceptual framework has the advantage of demonstrating that it is possible to delegate lower levels of autonomy to technological systems without threatening patients’ moral autonomy. Hence, ethically acceptable implementations can be identified. However, and this is the final aspect of the paper, such a conceptual framework needs to be operationalized, i.e., related to agency, so that the ethical claims can also be empirically tested. Here, four use cases are discussed and assessed to operationalize the framework.

## Agency and autonomy

The use and implementation of such devices is often highly disputed in practical ethics. Nevertheless, they seem to offer at least therapeutic benefits for persons with, e.g., Parkinson’s disease. That is why it is indispensable to develop a conceptual framework that allows a description of the interactions or even “co-actions” [21] of the system and the human agent. The effects of brain-computer interfaces on patients are assessed empirically in studies and hypothesized on a more speculative level via thought experiments. Schönau et al., proposed a model to operationalize dimensions of agency, such as responsibility and privacy. (Schönau, et al. [43]) This model assesses the conceptual background of human agency through empirical studies. Nevertheless, it is inchoate if it is not related to autonomy because it is widely assumed that autonomy is not only a characteristic that the human agent has but also the technological system (at least to a certain degree). Hence, a more complete model needs to be developed by classifying autonomy at different levels (operational, strategic, and moral). Technological systems can only operate on the first two levels because their behavior is based on dispositions within the scope of alethic modality, whilst human agency is based on deontic structures that are expressed via normative attitudes and statuses that cannot be reduced to alethic modality. Brandom, [10] This crucial difference can be used to develop methods for evaluating the ethical side of BCIs.

### BCIs and research methodology – agency

The primary focus of assessing the ethical and psychological effects of BCIs is on patient testimonials about their subjective experiences. The assessment tools are surveys to collect patient experiences (qualitative observations). It is thereby crucial to determine the criteria and indicators for the observables [22] and to establish a questionnaire that can yield a certain kind of “generalizability” of the results. (Flick, [12], pp. 114, 398, 495)^3^ Another important aspect to consider is that a patient might understand a concept such as autonomy differently from the investigator, because one focuses more on independence and the other on self-determination. Therefore, disambiguation is essential when analyzing the patients’ answers. The interpretation of the testimonials can also be problematic if other dimensions or concepts not part of the study are introduced. Kraemer discusses a case in which she assumes the importance of the patient’s decisions had less to do with autonomy than with authenticity. Kraemer, [31] Glannon, on the other hand, discusses the case in relation to autonomy. Glannon, [15] Such a discrepancy should be avoided and is also somewhat speculative. For instance, Kraemer’s conclusions are based solely on the testimony of one patient.

Another point that needs to be considered is that the ethical dimensions (e.g., responsibility, privacy, trust, authenticity) of “agency are intricately inter-related in an individual’s experience of agency. Questioning one’s responsibility, for instance, may lead to feelings of confusion over authenticity”: “Did I really do that? Is this me?” (Schönau, et al. [43], p. 173) Schönau et al., present an “Agency Map” that serves as a framework for translating dimensions or values into criteria. (Schönau, et al. [43], p. 178) This is similar to the specific abilities assigned to more general abilities in Jean Piaget’s theory of development. Piaget, [39] Rather abstract abilities or dimensions need to be specified in exemplary abilities (criteria) that allow for testing behavior, i.e., either measuring it (quantitative observables) or assessing it (qualitative observables). It is supposed that the exemplary or specific abilities (concepts) are equivalent and can be interchanged. Schurz, [45] Schönau et al., specifically write that their approach is to tie the dimensions “to a particular power (i.e., an agentive competency)”. Additionally, they generated questions for each dimension that serve as indicators, making the criteria (and dimensions) observable. (Schönau, et al. [43], p. 173 and 182)

Authenticity can then be described as a certain kind of persistence over time: “through achieving continuity via the integration of previous, future, and current states of the self.” (Schönau, et al. [43], p. 178) Authenticity can also be related to responsibility, i.e., feeling responsible for past, present, and future decisions or actions. Nevertheless, authenticity can also come with a certain kind of dissonance that the agent acknowledges as part of her personality. A person can, for example, recognize that there are good arguments against eating meat but still eat it. This “irrationality” is part of an authentic self and not directly a pathological state. It, therefore, depends significantly on how the indicators model the search space. Privacy can be specified “by negotiating with others over their access to the individual,” trust can be specified by how someone interprets “sensory feedback regarding one’s positioning,” and responsibility can be specified by how one can exercise “intentional control over a goal-directed action.” (Schönau, et al. [43], p. 178) Of course, these indicators only specify certain aspects of the more general concepts. Nevertheless, the approach of Schönau et al. describes how agency in BCI research can be assessed. Here, it is claimed that autonomy is foundational to agency. Hence, evaluating patients with BCIs needs, on the one hand, to be tied back to autonomy but, on the other hand, also integrated into human agency to make it empirically accessible. The advantage of the approach from Schönau et al. is the operationalization of various dimensions of agency.

### The foundational role of autonomy for agency

All four dimensions or concepts from Schönau et al., are related to the notion of autonomy, which encompasses different meanings, such as “independence”, “self-dependence”, and “self-determination”. (Gottschalk-Mazouz, [17], p. 238) Exercising control is also entailed by self-determination (“Do you feel that the (device mediated) movements you perform are under your intentional control?” (Schönau, et al. [43], p. 182), negotiating access is entailed by being independent (to a certain degree), and fostering self-trust is entailed by self-dependence (“Do you trust yourself when you are using the device?” (Schönau, et al. [43], p. 182). Nevertheless, there are overlaps in this classification. Still, it is essential to recognize that autonomy is the conditioning factor, as many issues revolve around who is the originator of the action: the device or the person/patient.

Schönau et al. mix different meanings of autonomy (self-determination and independence) when they state that “autonomous agency requires action that is self-governed (i.e. not subject to undermining influences […]).” However, such conceptual incompatibility is problematic. Furthermore, they claim that their focus is mainly on agency because of “its foundational relationship to autonomy” and

“[a]s such, agency is a necessary condition for autonomy, but it is a lower level phenomenon, i.e., part of what makes autonomy possible […]. We want to call attention to the reality that neural devices can alter our very capacity to act – to make choices and enact our intentions on the world – in ways that precede questions about whether or not those actions are autonomous.” (Schönau, et al. [43], p. 174).

The foundational relation is quite the opposite: autonomy is the basis to understand agency, not only because its different meanings are present in the different dimensions of agency discussed by Schönau et al., but also because any performance or behavior of a person can only be called an action if the agent is able to set herself in relation to this performance. Otherwise, it would just be some behavior that was elicited by external or internal factors. For example, iron reacts with oxygen in the presence of water as a catalyst. Sugar dissolves in water under the right circumstances. Objects have dispositional properties that result in a particular reaction or behavior.

However, some objections might be raised. It has been pointed out that one might intentionally act without doing it autonomously.^4^ For instance, a good person who has been brainwashed to do something bad would perform the actions intentionally, indicating that she has specific beliefs and desires while committing the bad deeds. Nevertheless, one would not ascribe to her being morally responsible for her deeds. Mele, [36] Claiming that this action has been performed non-autonomously would mean taking moral autonomy as the sole type of autonomy. At both the operational and strategic levels, the person acts intentionally and autonomously. Who else would act? Of course, from a more distant vantage point, one could claim that she behaves in a way that was elicited by the brainwasher. – This also has implications for the application of BCIs as discussed below when a grave personality change in a patient might occur. Despite the change, she still acts intentionally and autonomously, which makes it challenging to determine authenticity and responsibility.

In some circumstances, one might be forced to do something and, therefore, not act autonomously. For instance, when a robber threatens someone to hand over the wallet. Nevertheless, it seems that the action would be intentional, i.e., accompanied by beliefs and desires, although contrary to the values of the victim. However, the example can also be explained in terms of operational and strategic autonomy. Reacting either by handing over the wallet immediately or running away would be a reaction elicited by the circumstances and/or feelings of fear. Strategically evaluating the situation and considering different outcomes leads to an autonomous action at a strategic level. One could gamble and claim not to have a wallet or money. Moral autonomy and responsibility do not play a role in this example. The robber has one-sidedly revoked morality. Hence, operational and strategic autonomy do not undermine moral autonomy; rather, they are integral to our actions.

There are numerous actions in our daily lives that we perform that are neither morally relevant nor involve moral autonomy. Many of the actions on both operational and strategic levels are performed automatically, requiring minimal mental effort.^5^ Denying that some level of autonomy would entail such actions would mean dissociating the actions from the self. “The, I think, must accompany all my notions/representations,” as Immanuel Kant stated. (Kant, [27], p. B 131/132) It is a transcendental claim. However, it also has implications that can be understood by empirical evidence in self-testimonies of patients with Parkinson’s disease who have undergone DBS. They state that: “I feel like a robot” and “I feel like an electric doll.” (Schüpbach, et al. [44], p. 1813) This has been taken as a “threat to agency – the ability to make informed and rational choices – when a person’s actions do not flow from her intentions or beliefs but rather are the result of direct brain manipulation.” (Baylis, [2], p. 524) Nevertheless, dissociations can only be experienced if an autonomous self is presupposed.

We can therefore distinguish between minimal-intentional, autonomous, and morally responsible agency.^6^ Animals are directed toward objects (food, obstacles, etc.). They are performing minimal, yet intentional, actions.^7^ Autonomous agency presupposes a kind of self-awareness and comprehension of one’s own plans and how they can be adapted. Animals and technological systems can function on operational and strategic levels of autonomy; however, they cannot function on a moral level, since they lack responsible moral agency. Tomasello has demonstrated in studies and argued for a “shared intentionality” in humans, which is absent in apes (Tomasello [51, 52]). They cannot understand the intentions of others, which human children at a certain age already can, and which seems to be the basis for reciprocal recognition and reaching the level of moral autonomy.

### Models of autonomy in BCI research

Even though animals and humans react to certain stimuli, at least the latter can set themselves in relation to the stimuli. Gottschalk-Mazouz points out that autonomy cannot be reduced to mere reactions to something; instead, autonomy should be understood as the ability to actively set oneself into a relation to something: thoughts, attitudes, claims, systems, actions, persons, ideas, etc. (Gottschalk-Mazouz, [17], p. 238) In contrast, Cabrera et al., model autonomy based on the aspect “informed consent” ([11], pp. 37–39), following Beauchamp and Childress. (Beauchamp and Childress, [3], p. 77/78) They emphasize that the mood and affect strongly influence the “decision-making capacities,” particularly in the case of brain stimulation of patients who are in conditions such as psychiatric disorder, and that even the stimulation might come with mood changes to which patients with psychiatric disorders are also more prone. (Cabrera et al., [11], p. 39) Such influences on autonomy need to be investigated empirically to obtain a more realistic picture of informed consent, as a fully cognizant form of autonomy on the part of patients cannot be presupposed. However, such a decision-making capacity also needs to be differentiated into levels of autonomy. Otherwise, aspects of autonomy such as informed consent will always be weighed against a gold standard of fully cognizant or moral autonomy. For instance, a cardiac pacemaker functions on an operational level of autonomy (see below) and does not compromise moral autonomy. Nonetheless, a pacemaker does not come with changes in personality traits. It is also difficult to say how and when informed consent should be obtained, i.e., during stimulation or when it is turned off. (Cabrera et al., [11], p. 40/41) Therefore, further research on these issues is necessary.

Friedrich et al., model autonomy as a “multi-component ability.” The three abilities are as follows: (1) using “information and knowledge to produce reasons,” (2) ensuring “that intended actions are realized effectively (control),” and (3) enacting “intentions within concrete relationships and contexts.” (Friedrich, et al. [13], p. 19/20) Again, autonomy is understood regarding the gold standard of full cognizant self-rule, -government, and -determination. The first ability aims at having sufficient information and knowledge to understand situations, one’s own actions, preferences, or options at hand, and to produce reasons for actions, beliefs, etc. For a person, it is thus central to know “if she is a victim of self-deception, or if other autonomy-enabling and autonomy-inhibiting external circumstances are present.” According to Friedrich et al., this point is also crucial for the other two abilities. (2) There might be internal factors or influences that inhibit autonomous actions. For instance, in the case of mental disorders such as obsessions, the person might not have control over her actions. (3) Additionally, there might also be external factors that inhibit autonomous actions. For example, the lack of physical resources might inhibit the enactment of intentions. (Friedrich, et al. [13], p. 20/21) Consequently, the model of autonomy is based on the notion of being fully cognizant of such internal or external factors.^8^ However, most of our decisions, actions, and thoughts are not of this sort, but rather on the level of operational and strategic autonomy that entails certain forms of automatization. Furthermore, the question is: do we sometimes act solely in accordance with (moral) principles based on our free will (actions), and at other times solely on natural desires or inclinations (behavior)? Is it such an exclusive disjunction, i.e., that only one of these options is true, or is it an inclusive disjunction, i.e., that both options can be true? Do we sometimes act freely and sometimes based on our natural desires or external stimuli? These issues are discussed in action theory and in metaethics under the opposition of “internalism” vs. “externalism” for moral motivations. Internalists claim that a specific subjective motivation always needs to be involved, i.e., that a moral principle or reason can only be realized through an acting subject that is immersed in the world and that has motivations and interests (see, e.g. [5]. The strict opposition of an exclusive disjunction seems rather absurd. In actions, a motivation, interest, inclination, or desire is always present. Still, we can also set ourselves in relation to our interests and recognize moral reasons as guiding principles of our actions. Actions can only be realized through our interests and motivations (this is the *voluntative* element of autonomy). This also means that autonomy is not only a cognitive task or property but can only be ascribed to someone who performs actions based on a voluntative element (desire, interest, etc.). A machine cannot refuse to act because it desires something else.

### Types of autonomy

A performed action can be interpreted in two (extreme) ways: either as an altruistic action or as a selfish action (that someone only helps to receive help or to get something in return). It cannot be read from the actions performed or the words uttered whether someone is trustworthy or doing something for moral reasons. It is based on an act of recognition in the sense of acknowledging someone as morally good, rather than cognizing their inner functioning. There are no criteria to cognize the freedom or the moral reasons of a person, but we can recognize or acknowledge them as the guiding principles of her actions. Autonomy is therefore not based on a property or a list of properties that one must have to count as an autonomous being. It is also not a sole act of recognition that I only recognize you or myself as autonomous, but a reciprocal action that can only be performed by at least two beings that recognize each other as autonomous. Brandom, [9] To recognize something or an intelligent system as autonomous can only come, therefore, in degrees of closeness to this reciprocal relation, but this also sets some limits for the responsibilities that can be delegated to the systems. To clarify such degrees of closeness in human‒machine interaction, three types or levels of autonomy can be differentiated [23] (see Table 1).

**Table 1:** This table provides a summary and translation of Christoph Hubig’s description of the types of autonomy. (Hubig, [23], p. 282/283)

Operational Autonomy	Strategic Autonomy	Moral Autonomy
Systems have certain degrees of freedom to select the means for achieving an objective, thereby improving the effectiveness and efficiency of an action. Driver-assistance systems (in the car) have, for instance, operational autonomy.	Systems have certain degrees of freedom in the selection of the optimal ends or objectives within a general framework of goals. For instance: prioritization, duration, or deviation, etc. An autonomous vehicle could be given the goal of reaching a destination, and the system can select a detour to get there faster, because there is more traffic on the shorter route. This is an example of prioritization, duration, and detour.	Moral autonomy is based on freedom, where one not only knows the goals but also recognizes the goals and principles: “I recognize the principle as a reason for my action.” Recognition is attributed to oneself, i.e., a subject recognizes herself as a person.
Responsibility at the operational level can be almost completely delegated to systems within certain limits.	Responsibility at the strategic level can be partially delegated to systems, as long as human interventions are possible and options are available to interrupt or stop the processes.	The responsibility of this type should not and cannot be delegated to systems, because technological systems are not capable of self-attribute recognition (although they may be able to form representational knowledge about themselves). Ethics is oriented here on the ideal of human dignity and based on human rights.

Moral autonomy is expressed in a normative and deontic vocabulary (commitment, entitlement, responsibility, (moral) norms). Brandom, [8] Operational and strategic autonomy, in contrast, can only be expressed in alethic modal vocabulary. Technological systems might be able to choose from available possible means or ends that form a realm or space of possibilities. This could be expressed as reactions of dispositions that they possess and in counterfactual statements: “If systems S had chosen means M, it would have resulted in output O.” Such dispositions of intelligent systems express “good” discriminations or inferences (“*ranges of counterfactual robustness*”). (Brandom, [10], p. 103) Consequently, they function well when they reliably reproduce effects.

Normative vocabulary cannot be reduced to alethic modal vocabulary. Even though in virtue ethics and metaethics some approaches use modal vocabulary to describe virtuous behavior (see, e.g., [19, 40, 47, 41]. It underdetermines human agency because it does not do justice to its normative dimension. Brandom offers an extensive analysis of the relation of modal and normative vocabulary and aims to show “how normative vocabulary can serve both as a pragmatic meta-vocabulary for modal vocabulary and as the basis for a directly modal formal semantics for ordinary empirical vocabulary that does not appeal in any way to a notion of truth.” ([10], pp. 116, and 92–116) He states:

“Normative concepts make explicit commitments that are implicit in any use of concepts, whether theoretically in judgment or practically in acting intentionally – that is, in endorsing practical maxims. Judgment and agency are implicitly normative phenomena because they consist in the application of concepts, and applying concepts is undertaking commitments and responsibilities whose content is articulated by those concepts. (For Kant, specifically moral normative vocabulary makes explicit commitments that are already implicit in the practical use of concepts to endorse maxims, ends, and plans.)” ([10], p. 110)

Thus, the interaction within a normative practice between humans is different from human-system interactions. The dispositions that are formed by the intelligent system are based on tracking users and the expectations that the users have about the system. However, the relation or interaction is one-sided and asymmetric because the users usually perceive themselves differently from how the system “perceives” them, and the information basis and the functioning of the system are usually opaque for the user. In contrast, the interaction between user and developer yields a process of reconciling or aligning expectations of expectations (*Erwartungserwartungen* [34]. Hubig, [24] This is in line with the difference of reciprocal relations of recognition between persons and the counterfactual robustness that a system can demonstrate by its acquired disposition (may they be learned or programmed) to interact with persons.

The above-mentioned types of agency might provide a direction for integrating the three different levels of autonomy. Each level presents a partially independent capacity since each level can be performed independently but presupposes the other in developing it; in a way, they are therefore also progressive. Strategic behavior presupposes some kind of operational adaptability, and moral judgment presupposes strategic autonomy, such as differentiating, ranking, modifying, and prioritizing goals, or intruding sub-goals. Moral autonomy represents a qualitatively different normative status which cannot be reduced to dispositional functioning.^9^

The traceability of the effects of behavior from hybrid agents, such as a person with an invasive brain-computer interface, is even more difficult. It might not be possible to abductively infer who or what initiated the behavior or an emotional state. It might be possible to infer from the effect/behavior to the system or the human agent as an initiator of the action only if research enables one to make this distinction. For this purpose, it needs to be established which operational or strategic behavior is based on the dispositional features of the system and which operational or strategic behavior is based on human agency. This may not be transparent or possible for the human agent with a BCI. Still, it should at least be possible for researchers and developers to determine the dispositional functioning of the systems. Humans are also not always fully aware of what causes their behavior and whether they initiate it voluntarily. Requiring such transparency as a condition for hybrid agency would overestimate the rationality of human agents and the possibilities of self-reflection, even in purely human agency.

## Assessment of autonomy regarding BCI research

Although the topic is widely discussed in various fields, ranging from medicine to the philosophy of technology and biomedical ethics, the underlying modal and conceptual structure has not been sufficiently scrutinized. Technological devices react to stimuli based on mechanical or computational processes that are sometimes enhanced and optimized by artificial intelligence. To operationalize the conceptual structure, four use cases are described, taken from examples in the literature. Gilbert, [14], Glannon, [15], (Kellmeyer, et al. [29], Kraemer, [31], Schönau, et al. [43]) Some are real cases, and some are thought experiments, but they are based on recent developments in the area of BCIs. The cases regarding the types of autonomy are discussed. This has the advantage of operationalizing and testing the concept. Usually, these cases are discussed under one primary value or concept (e.g., authenticity [14], but mostly, the implementation of technology comes along with conflicts of values (e.g., safety vs. accountability). Thereby, the types of autonomy should help to clarify these conflicts.

It is ethically acceptable to delegate operational or strategic autonomy to a BCI if the patient’s autonomy is preserved or increased. For instance, speech output for someone who lost the ability to speak can be optimized via AI and an integrated chip in the brain. It increases the patient’s autonomy while delegating operational autonomy to the system. However, task delegation can become problematic if the system chooses, changes, or prioritizes goals in ways that the patient cannot comprehend, assess, challenge, or interrupt. For example, a patient with a BCI feeling alienated from herself, hence not authentic anymore, would start choosing goals that she cannot comprehend and that are not coherent with former life choices or plans.

### Use case 1: Depression – well-being and context

Adaptive or closed-loop DBS is one of the promising technological improvements to treat Parkinson’s disease (Swann, et al. [49]) or major depressive disorders (MDD) [54]. Also, Schönau et al., apply their “Agency Map” to three fictional case studies (Schönau, et al. [43], p. 179) that are nonetheless based on existing technologies and clinical research to discuss the capabilities of such devices and the consequences of their use, in one case about a woman whom they named Cora, they discuss the use of a deep-brain stimulator for the treatment of a major depressive disorder: The device records her brain activity and stimulates certain areas of the brain. “She, and they [her friends and family], are optimistic that the device will allow her to get back to the Cora they remember.” (Schönau, et al. [43], p. 181) The device would then also be able to “automatically” adjust and treat her disorder. In the case that Cora would feel “reliant on her own device,” she would not need “to take an active role in dealing with her condition” anymore. (Schönau, et al. [43], p. 181) Consequently, the device can work autonomously, and it will no longer be possible for Cora to experience who or what is the originator of feelings or actions. Nevertheless, in some situations, it is appropriate and normal to feel sad, for example, when attending a funeral. How can the device “know” in which context the person is, and which kind of reactions are appropriate? (Schönau, et al. [43], p. 181)

As a means to address this problem, the different dimensions of autonomy need to be discussed, how they are implemented in BCIs, and the limits of the dispositional functioning of such devices need to be considered. Users of technological devices can have expectations about how the device should function and how they would use it. Additionally, developers can have expectations of how the device will be or should be used. The expectations of both groups are aligned in the research and development phase of a device. It is based on relations between persons (developers and users) that align their expectations and interests. In contrast, such an alignment procedure cannot describe the human-system interaction. Autonomous systems collect user data by tracking or monitoring them, thus creating relevance rankings, establishing routines, or ascribing roles to the user (or stereotypes). A movie recommendation system, for example, tracks users and makes recommendations (relevance rankings). All the collected data and information produce a virtualized context. However, such a context is underdetermined. A smart home that should detect when an elderly person falls on the floor and might need help could also detect similar impacts when grandchildren visit and jump around. It might even be the case that it wrongly calls an ambulance. The modeling of smart or virtualized environments must always be recontextualized. (Hubig, [21], pp. 134–145) Thought experiments that imagine counterfactual situations can point out the one-sidedness of models before devices are implemented, as in this first use case, when a person with depression attends a funeral. It is a context where it is okay to feel sad and depressed. The situation or context of the lifeworld is overdetermined, i.e., it carries information, meanings, and determinations that are not captured by smart contexts because they are always modeled based on a certain range and finite number of conditions.

Here, the device is not only working on an operational level. It makes strategic decisions within the user’s lifeworld; however, without context-sensitive information. This threatens the user’s moral autonomy since she cannot understand her behavior in certain situations. When designing such devices, the user should be in the loop and asked before it stimulates the brain. Nevertheless, this also seems impractical when it must do it several times a day. In general, the strategies within the systems need to be transparent to users; otherwise, it can lead to situations where users do not understand what might be happening to them. However, this might also threaten the user’s moral autonomy since it makes her responsible for understanding how the device functions under different life situations and how it affects her behavior. Furthermore, the person suffering from depression must be willing to activate the device. Building such systems in a context-sensitive way might also be challenging since it seems rather difficult to distinguish, also on a neurobiological level, between clinical depression and normal sadness (Arias, et al. [1]). Furthermore, the context is also not determined within psychiatry because the “Diagnostic and Statistical Manual of Mental Disorders” (DSM-5) from 2013 includes bereavement for diagnosis of major depression, which was excluded before (Arias, et al. [1]). Even on the psychiatric diagnostic level, the criteria and indicators for depression are contentious. How, therefore, can a context-sensitive device be developed? Hence, the user should be in the loop since it increases her strategic autonomy, as long as she can understand how the device functions in certain situations.

### Use case 2: ALS – control and privacy

Certain devices can preserve or regain personal autonomy by enabling control for patients with neurodegenerative diseases such as amyotrophic lateral sclerosis (ALS). “Neurodegeneration in ALS in later stages results in partial or complete loss of all voluntary motor control – the locked-in state, which limits or prevents communication by natural means. A BCI device may thus enable or preserve communication in these patients.” (Kellmeyer, et al. [29], p. 624) This can be achieved by using machine learning to help select the letters. Kellmeyer et al. thus claim that “[a]t present, in our view, the degree of system autonomy of the available BCI solutions strengthens rather than undermines personal autonomy by enabling otherwise severely impaired patients to regain control of communication and movement to some degree.” (Kellmeyer, et al. [29], p. 628) Kellmeyer et al. call it “shared autonomy” (Kellmeyer, et al. [29], p. 626/631), but the system is not fully autonomous, even though it is built with machine learning algorithms, because the system does not make decisions; otherwise, it would choose the letters. It only reacts to the patient’s input, which is then used to train the system and improve its performance. This is, therefore, more of an assistive system that functions at an operational level of autonomy, yielding an increase in the patient’s personal autonomy or control.

Nonetheless, it appears to pose a threat to the patient’s privacy. One could imagine a case in which the system detects and outputs thoughts of the person that others should not hear. This hypothetical situation goes beyond the operational level of such systems. However, on the operational level, data collection does not pose a direct threat to the patient if sufficient security measures are taken to prevent data leakage, such as through hacking. Assistant systems, while driving, also collect an increasing amount of data on the driver, such as eye movements, to measure the level of the driver’s tiredness. Companies must be careful in how they handle and secure data. This is more of a legal aspect covered by existing laws; however, ethical issues might also arise since even if no personal data is collected, inferences about the user can be made that can be considered an intrusion into their privacy, as for instance on social media platforms where the usage can reveal gender, age, etc., even when it is not explicitly stated in the profile [37]. Nonetheless, it is essential to clarify the concept of autonomy. To speak of “shared autonomy” obscures more important distinctions within human-system interactions. Furthermore, a concept like invasiveness can be perceived differently; for instance, “people recognize several kinds of invasiveness: physical, emotional, and lifestyle” [7]. Hence, collecting data to regain control (autonomy) might also be perceived by patients as an intrusion into their privacy (inner thoughts) and thus raise issues regarding the moral level of autonomy. The collection of the data should thus be transparent to the patient, i.e., who has access to the data, what this access looks like, to what extent the patient can withhold private thoughts when using the device, etc.

### Use case 3: Epileptic seizures – safety and accountability/responsibility

Additionally, in the case of predicting epileptic seizures, a BCI could increase control and independence. (Gilbert, [14], pp. 5–7) In this case, however, a certain kind of “shared autonomy” can be assumed because there “will be no need for the patient to consent to each occurrence of the therapeutic activation; the device will operate ‘autonomously.’” ([14], p. 8) However, the question would be whether the patient would perceive it also as a threat to her autonomy or as something to gain control and improve her quality of life. If the person is in the loop, she still has some autonomy in the decision-making process. This can be done through some output from the system that shows that there might be, for example, a seizure, and she can, therefore, either cease certain activities to be safer or decide to use medicine. While it is an advantage to enhance the strategic autonomy of the patient “with respect to her seizures” by giving “the patient as much knowledge about the state of her system as possible,” it also has “the disadvantage of requiring the patient to act in a way that maximizes safety.” (Kellmeyer, et al. [29], p. 627) Kellmeyer et al., point out that standards and “[c]riteria for distinguishing when human individuals should be able to override medical devices and when medical devices should intervene to override human autonomy remain to be developed.” (Kellmeyer, et al. [29], p. 627).

The person who is in the loop has more strategic autonomy but also more responsibility. (Kellmeyer, et al. [29], p. 630) In the case that the person is out of the loop, it decreases her autonomy, and a particular form of “informed consent” in the beginning is needed. (Gilbert, [14], p. 8) Even if the system works effectively and improves the patient’s situation, it is essential to have the option for the patient to deactivate the system or disengage from it at any time. (Gilbert, [14], p. 9) The automatic functioning of the system (for example, administering medicine when a seizure is detected) can be situated at an operational level of autonomy similar to Advanced Driver Assistance Systems, where responsibility can be delegated to the system if it functions reliably and has been tested for safety. Nevertheless, such functioning enhances the patient’s strategic autonomy, consequently increasing her responsibility regarding how she manages her disease.

The ethical implications surrounding these issues require further discussion. For instance, the device should automatically intervene when a decision must be made quickly, and the patient would not have time to gather all the information. This is particularly important in situations where the safety of the patient and others might be at stake. The patient, however, should be able to override or prevent the intervention when she can gather sufficient information to make a well-informed decision and understands the consequences. Deliberated judgments in this case take place within the trade-off between safety, autonomy, and responsibility, where it is important to clarify who will be held accountable in the end: the patient or the manufacturer. Hence, even apparent issues at the level of strategic autonomy ultimately lead to ethical issues regarding moral responsibility.

### Use case 4: Parkinson’s disease – identity and informed consent

Furthermore, difficulties arise where the functioning of a device changes the behavior or personality of someone:

“In a recent article in the Dutch medical journal Nederlandse Tijdschrift voor Geneeskunde, Leentjens et al report a case study, […] in which a 62-year-old patient with Parkinson’s disease faced a dilemma as a result of DBS. The patient who had a brain pacemaker implant and was under treatment with DBS, developed, along with other psychological and social problems, a permanent manic state that could not be controlled via medication.” ([31], p. 757)

In the manic state, when the device was switched on, “he was not believed to be accountable and rational, meaning, unable to give informed consent. In the terminology of medical ethics, he was considered to be mentally incompetent and not legally accountable, that is, he was not an autonomous agent.” ([31], p. 757/758) Kraemer uses “autonomous” and “mentally competent” as synonyms. (Kraemer, [31], p. 758) She discusses the case under the heading of authenticity ([31], p. 757), but this is speculative because it is based on her interpretation of the case rather than the patient’s testimonials. (She writes, for instance, “It is at least a possibility that he could have decided to stay switched-on because he felt authentic in the stimulated state and because he regarded his unstimulated life as alienating.” ([31], p. 758) Glannon, on the other hand, discusses the same case under the heading of autonomy ([15], p. 290): “Paradoxically, an autonomous decision to consent to a medical treatment would make him lose his autonomy and capacity to subsequently choose to continue this treatment and to have or forego others.” ([15], p. 291)

A testimonial from the patient would be needed for authenticity. It is problematic when such a discrepancy in interpretation exists. However, the case seems to pose an ethical conundrum which cannot be dissolved: a patient might autonomously decide to get DBS treatment. Even if the patient would not fall into a manic state but change radically in his personality, how should we consider his capacity for making autonomous decisions? Just because he might treat everyone badly and feels “authentic” doing so might not justify forcing him to remove the BCI. Patients’ provisions could help to some extent and are adequate means when someone irreversibly falls into a coma. We could imagine a patients’ provision for cases regarding BCIs as enhancing the strategic autonomy of the patient when they cannot make decisions anymore, as in an untreatable manic state. However, in cases where “only” the personality changes, removing the BCI would seem far too intrusive on the patient’s autonomy, even if they, before the treatment, stated that it should be removed in cases of grave personality change.

Here, empirical studies are needed to obtain a more complete picture. For instance, the types of autonomy could be operationalized in qualitative research to include patients’ perspectives to better understand the intricate interplay between authenticity, autonomy, and responsibility. Bluhm et al., have criticized the philosophical literature’s focus on extreme behavior following DBS and personality changes. They propose an approach drawn from the “empirical literature on patients’ experiences” and based on a “relational narrative framework” to enhance the understanding of the self and the more common issues that patients face during adjustments. Bluhm et al. [6]. However, the relationship between autonomy and informed consent to or the refusal of treatment has at least practical consequences, leading to questions about who is or should be held accountable and how the patient’s ability to give informed consent can be assessed. Cabrera et al., point out that “for many current and future practitioners of brain stimulation,” this “may represent a new clinical skill set that will need to be acquired.” (Cabrera, et al. [11], p. 39)

## Conclusion

### Implications for empirical research

Kellmeyer, [29] state that in the case “when human subjects and (semi)autonomous systems work in concert,” it is important to consider “moral and legal accountability”. (Kellmeyer, et al. [29], p. 629) Values such as transparency (of the system), autonomy, and accountability must be balanced. Still, the criteria and indicators must be developed to assess the situation and make the evaluation manageable and transparent. The “Qualitative Agentive Competency Tool (Q-ACT)” of Schönau et al., is a step toward this. (Schönau, et al. [43], p. 182) In the discussion of the case of the Dutch patient, no indicators are visible that help to evaluate the case, and it is only one patient. This suggests future research directions to clarify which values are at stake and how processes can be established to operationalize them. This can, of course, not be achieved by solely relying on expert advice from ethicists or medical professionals. Kellmeyer et al., recommend “a political decision making process that is guided by working models of Jürgen Habermas’ theory of ‘deliberative democracy.’” All stakeholders should be involved in “this bottom-up model of political legitimization, extensive public discourse, dialogue, and deliberation” to establish regulations. A “top-down ‘expertocratic’” model would, on the contrary, impose regulations “without adequately consulting the needs of the most vulnerable stakeholders,” particularly in the case of BCIs “the severely neurologically impaired patients and their families.” (Kellmeyer, et al. [29], p. 630/631) Even though it is an important task to involve all the stakeholders and consult the patients and their families, it implies that they are enabled in a certain way to participate in that discourse. Still, due to the depth of engagement of this technology, it is a double-edged issue: on the one hand, BCIs enable independence and self-determination, but on the other hand, they can also make personal autonomy impossible.

The dispositional functioning of devices is not so pressing when the device is assisting the person, for example, in the use case where machine learning algorithms help to select letters to enable communication for persons with ALS. It is an assistance system that functions at an operational level. However, cases become more complicated when the situation or context plays a significant role. Here, developers need to anticipate counterfactual situations in thought experiments, i.e., situations where the device functions in accordance with the range of its dispositions, regardless of the context of the lifeworld, because it is not part of its artificial environment. For example, attending a funeral not only engenders but also calls for feelings of sadness. The environment of the devices is created by tracking the person’s neural activity, not her attendance at a social event. Consequently, the modeling of dispositional properties and the types of inputs for devices needs to be analyzed and tested in different situations. Furthermore, it needs to be established to what degree systems can or should have operational autonomy, i.e., to choose certain means for the user, and to what degree systems can or should have strategic autonomy, i.e., to select ends for users. The dispositional functioning of systems can be tested, as is done, for instance, in automotive research for integrated safety systems.

### Ethical aspects

The (ethical) assessment of such devices is mainly done by qualitative research, interpretations of a few cases, and thought experiments. Conducting behavioral studies with control groups is challenging due to the diverse use cases and the limited number of users. Significant work remains to be done before a comprehensive understanding of hybrid agency is established. The present conceptual research tried to shed light on the normative nature of human agency and the dispositional nature of technological devices. Both aspects are still quite general and require further specification, as well as empirical studies that complement the conceptual framework. Such a “problem-induced technology assessment” is used to seek technological solutions for given societal tasks. In contrast, a “technology-induced technology assessment” evaluates technology already at the series-production readiness stage. (VDI, [53], p. 81/82) In the case of BCIs, any kind of technology assessment at the stage of series production readiness would be problematic due to the manifold challenges that this technology poses for our understanding of ourselves as humans. Within the range of a “problem-induced technology assessment,” the task of providing health care for patients with ALS or Parkinson’s disease can be managed through different technologies and medical treatments. Still, it can also meet different criteria to fulfill the value of health, such as enabling control or diminishing pain. This always needs to be specified by including all the stakeholders, particularly in the case of BCIs, “the severely neurologically impaired patients and their families.” (Kellmeyer, et al., [29], p. 630/631) Although enabling control via assistance systems is an advancement, the feedback and dissonances from the action environment are usually virtualized and system-immanent, such as in the case of the patient with depression who might not feel confused by attending a funeral in a good mood. Consequently, an exit strategy, such as an off switch for certain situations, might be helpful. It appears, at first glance, to present an ethical imperative to enable the human agent to act, judge, and abstain from using the system ([20], p. 234). However, even though the person in the loop might have “more” autonomy, they also have more responsibility. (Kellmeyer, et al. [29], p. 630) These are all ethical issues that require further investigation.

### Design and governance

The devices themselves cannot be held responsible. Human actors remain responsible: patients, clinicians, researchers, and developers. For that purpose, institutional frameworks must be established to help evaluate such devices across various fields and create ethical guidelines, as in the IEEE Brain initiative. To obtain a more fine-grained understanding, which is based on the tripartite model of autonomy, I suggest the principle that it is ethically acceptable to delegate operational or strategic autonomy to a BCI if the patient’s autonomy is preserved or increased. For instance, optimizing the operational functioning of a device via AI (e.g., for speech output) might not affect the moral level of autonomy. It increases the patient’s autonomy while delegating operational autonomy to the system, as long as the device functions safely and does not intrude into the patient’s privacy. Therefore, an increase in systems’ autonomy does not necessarily diminish human autonomy and may even be upheld on the operational level. On a strategic level, the patient might also defer responsibility to the device as long as she can comprehend, assess, challenge, or interrupt the device’s functioning. This has crucial implications for the design of such systems; while on the operational level well-being, safety, and privacy play a crucial role, questions of intervention on a strategic level must be considered: at what point is the patient in the loop, to what extent must they be informed about the functioning of the device and its applied strategies, etc.? Strategic autonomy demands more robust safeguards, and moral autonomy cannot be delegated to a technological system. Ultimately, moral questions might also arise. However, autonomy must be considered as a crucial foundation of developing governance structures such as institutional frameworks and ethical guidelines for evaluating BCIs and not focus solely on the various affected values (such as justice, safety, privacy, etc.), since without this guiding principle such guidelines lack clear direction.

## Data Availability

No datasets were generated or analysed during the current study.

## Footnotes

1 I am thankful to an anonymous reviewer for pointing out this distinction.

2 I am thankful to another anonymous reviewer for pointing out this structure.

3 For an overview of qualitative research methods, see (Flick, [12]), (Lune and Berg, [35]).

4 I am thankful to two anonymous reviewers for the examples.

5 This is a little similar to the distinction between system 1 and system 2 by Daniel Kahneman. (Kahneman, [26]) The differentiation of the two systems into operational, strategic, and moral autonomy might even yield a more fine-grained picture here.

6 I am thankful to an anonymous reviewer for this distinction.

7 Intention is taken here in the sense of directed toward, as in the phenomenological tradition from Husserl and Heidegger. However, it might also be claimed that intentions must be already semantically imbued, so only humans can perform intentional actions.

8 Friedrich et al. apply their model to “*passive* BCIs” and “*reactive* and *active* BCIs”. (Friedrich, et al. [13], pp. 22-26) This is similar to the here proposed strategy. However, they do not distinguish different levels of autonomy neither on the side of the human agent nor on the side of the technological system.

9 Arguing for such a non-naturalistic distinction goes beyond the scope of this paper. I do not think that patients assess their lived experiences based on this metaphysical theory. However, as a foundation, it can contribute to normatively evaluating BCIs. (I am thankful to an anonymous reviewer for pointing this issue out).
